# Resolvin D1, a Metabolite of Omega-3 Polyunsaturated Fatty Acid, Decreases Post-Myocardial Infarct Depression

**DOI:** 10.3390/md12115396

**Published:** 2014-11-13

**Authors:** Kim Gilbert, Judith Bernier, Roger Godbout, Guy Rousseau

**Affiliations:** 1 Centre de biomédecine, Hôpital du Sacré-Cœur de Montréal, 5400 boul. Gouin Ouest, Montréal, PQ H4J 1C5, Canada; E-Mails: kim.gilbert@umontreal.ca (K.G.); judith.bernier-ouimet@umontreal.ca (J.B.); roger.godbout@umontreal.ca (R.G.); 2 Département de pharmacologie, Université de Montréal, C.P. 6128 Succursale Centre-ville, Montréal, PQ H3C 3J7, Canada; 3 Département de psychiatrie, Université de Montréal, C.P. 6128 Succursale Centre-ville, Montréal, PQ H3C 3J7, Canada

**Keywords:** Omega-3, Resolvin D1, myocardial infarction, apoptosis, limbic system, depression

## Abstract

We hypothesized that inflammation induced by myocardial ischemia plays a central role in depression-like behavior after myocardial infarction (MI). Several experimental approaches that reduce inflammation also result in attenuation of depressive symptoms. We have demonstrated that Resolvin D1 (RvD1), a metabolite of omega-3 polyunsaturated fatty acids (PUFA) derived from docosahexaenoic acid, diminishes infarct size and neutrophil accumulation in the ischemic myocardium. The aim of this study is to determine if a single RvD1 injection could alleviate depressive symptoms in a rat model of MI. MI was induced in rats by occlusion of the left anterior descending coronary artery for 40 min. Five minutes before ischemia or after reperfusion, 0.1 μg of RvD1 or vehicle was injected in the left ventricle cavity. Fourteen days after MI, behavioral tests (forced swim test and socialization) were conducted to evaluate depression-like symptoms. RvD1 reduced infarct size in the treated *vs.* the vehicle group. Animals receiving RvD1 also showed better performance in the forced swim and social interaction tests *vs.* vehicle controls. These results indicate that a single RvD1 dose, given 5 min before occlusion or 5 min after the onset of reperfusion, decreases infarct size and attenuates depression-like symptoms.

## 1. Introduction

Myocardial infarction (MI) affects quality of life because of attendant cardiac limitations, while 15%–30% of MI patients incur depression [1]. Post-MI depression has major outcomes since the risk of mortality is increased by 4-fold within 6 months after MI [2]. However, despite this consequence, the mechanism of MI-related depression is poorly understood.

We developed an experimental model to study the question. Our data indicate that after MI, depression-like symptoms can be documented by converging evidence obtained with behavioral testing [3,4]. These symptoms can be attenuated by multiple interventions, such as antidepressants [3,4], probiotics [5], high-polyunsaturated fatty acid (PUFA) omega-3 diets [6] and pentoxifylline [7]. One potential mechanism that could explain the link between MI and post-MI depression is the inflammatory process triggered by the ischemic period [8]. For instance, tumor necrosis factor (TNF) and interleukin-1beta (IL-1β) are pro-inflammatory molecules common in both conditions, and reduction of their circulating levels could improve depressive symptoms [9,10,11].

Omega-3 fatty acid metabolites, recently identified and known as Resolvins, showed anti-inflammatory properties and pro-resolution of inflammation in different models [12,13]. Two series of Resolvins have been described: E series (RvE) derived from eicosapentaenoic acid (EPA), and D series derived from docosahexaenoic acid (DHA) [14,15]. EPA and DHA are metabolized to RvE and RvD by acetylated cyclooxygenase (COX)-2 and 5-lipoxygenase or through a COX-independent pathway involving cytochrome P-450 or 15-lipoxygenase [16]. Resolvins signal via activation of their G protein-coupled receptors: RvE signals through ChemR23 and BLT1 receptors, and RvD1 via GPR32 and ALX receptors [17,18]. Resolvins decrease neutrophil cell migration in inflamed tissues, promote phagocytosis of apoptotic cells, and anti-inflammatory properties by reducing the expression of inflammatory cytokines [19]. Previous work from our lab has disclosed beneficial effects of RvD1 in MI, reducing infarct size and neutrophil accumulation in the ischemic myocardium [20].

Since we have hypothesized that post-MI depression is related to the inflammatory process induced by myocardial ischemia and that RvD1 possesses anti-inflammatory properties, the present study was undertaken to determine if RvD1 could attenuate the depression-like symptoms observed in our experimental model by its administration before ischemia or 5 min after the onset of reperfusion.

## 2. Results

### 2.1. Infarct Size

**Figure 1:**
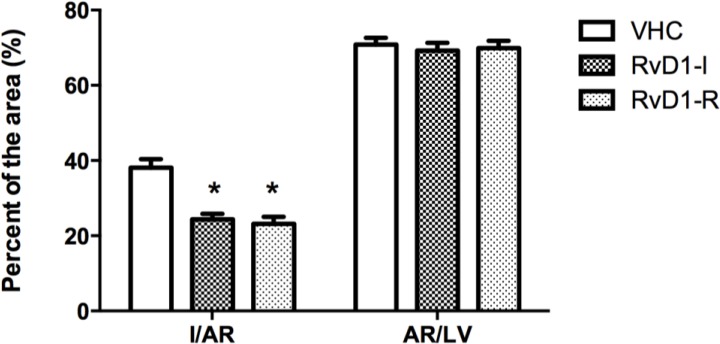
Infarct size (I) expressed as percentage of the area at risk (AR), and AR as percentage of the left ventricle (LV). Values are means ± SEM (12 rats per group). VHC = vehicle; RvD1-I = Resolvin-D1 administered 5 min before ischemia; RvD1-R = Resolvin-D1 administered 5 min after the onset of reperfusion.

MI size (I), expressed as percentage of the area at risk (AR), displayed significant between-group differences (F(2,33) = 18.99; *p* < 0.05). *Post hoc* analysis revealed significant differences between groups receiving 0.1 μg RvD1: before ischemia (RvD1-I) or after reperfusion (RvD1-R) compared to the control (vehicle) group. The AR, expressed as percentage of the left ventricle (LV), was similar between groups, representing about 70% of the LV (Figure 1).

* Indicates significant differences between the treated and vehicle groups; *p* < 0.05.

### 2.2. Behavioral Tests

#### 2.2.1. Social Interaction Test

**Figure 2:**
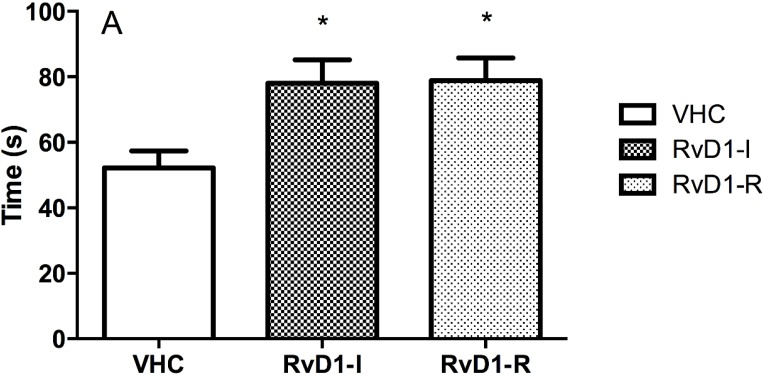

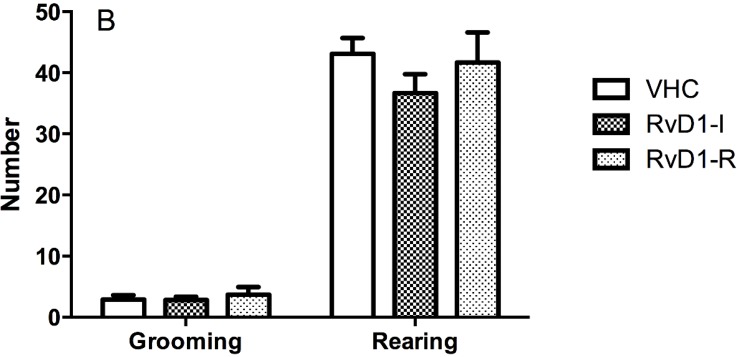
Social interaction test results on day 15 post-reperfusion. (**A**) Interaction time in seconds (s); (**B**) Number of groomings and rearings per group. Values are means ± SEM. VHC = vehicle; RvD1-I = Resolvin-D1 administered 5 min before ischemia; RvD1-R = Resolvin-D1 administered 5 min after the onset of reperfusion.

Analyses of social interaction duration disclosed significant between-group differences (*F*(2,25) = 5.30; *p* < 0.05). *Post hoc* analysis showed significant differences between groups receiving RvD1 compared to the controls (vehicle), indicating that treated rats were more interested in interacting with their congeners. No differences in grooming and rearing behavior were observed between groups (Figure 2).

* Indicates significant differences between the treated and vehicle groups; *p* < 0.05.

#### 2.2.2. Forced Swim Test

**Figure 3:**
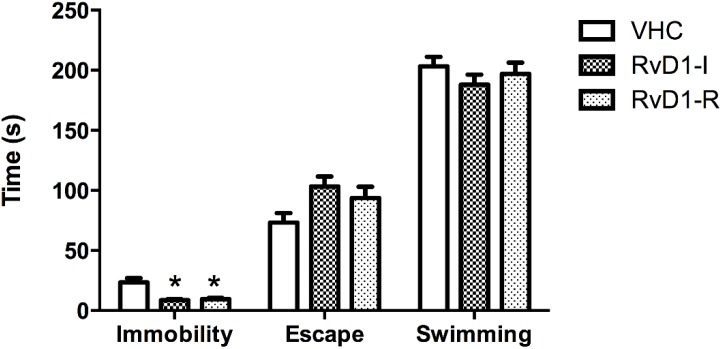
Forced swim test results on day 16 post-reperfusion. Immobility, escape and swimming are in seconds (s). Values are means ± SEM. VHC = vehicle; RvD1-I = Resolvin-D1 administered 5 min before ischemia; RvD1-R = Resolvin-D1 administered 5 min after the onset of reperfusion.

Results of the forced swim test revealed significant differences between groups for immobility time (F(2,15.3) = 8.05; *p* < 0.05). *Post hoc* analyses detected significant differences between groups receiving RvD1 compared to the controls (vehicle), indicating a potential antidepressant effect of RvD1in this test. No differences between swimming or escape time were seen between groups (Figure 3).

* Indicates significant differences between the treated and vehicle groups; *p* < 0.05.

**Figure 4:**
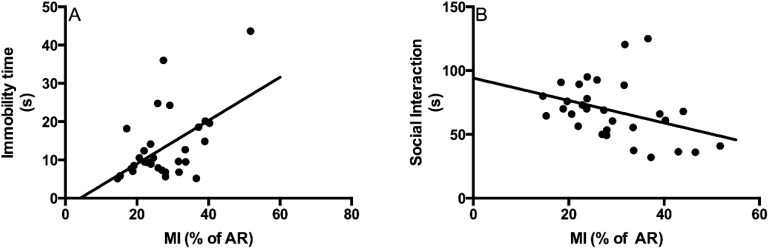
(**A**) Correlation between myocardial infarct size (% of the area at risk) and immobility time in the forced swim test. The results indicated a correlation between both parameters (*r*^2^ = 0.28; *p* < 0.05): a larger myocardial infarct size was accompanied by an increase in immobility time; (**B**) Correlation between MI (% of the area at risk) and socialization time. The results indicated a correlation between both parameters (*r*^2^ = 0.13; *p* < 0.05): a shorter socialization time was associated with a larger myocardial infarct size. MI: myocardial infarct.

Regression analysis between MI (% of the AR) and immobility time in the forced swim test or between MI (% of the AR) and the socialization time indicated a small, but significant correlation between these parameters (Figure 4). These results indicated that the modulation of infarct size can have an effect on performance in these behavioral tests.

## 3. Methods

### 3.1. Experimental Design

Forty 3-month-old Sprague-Dawley rats (Charles River Canada, St-Constant, QC, Canada), weighing 325–375 g at the start of the experiments, were studied. They were handled in compliance with regulations of the local animal care committee and in accordance with guidelines of the Canadian Council on Animal Care*.* All animals were housed individually, under constant conditions (temperature 21–22 °C and humidity 40%–50%), including a 12-h light-dark cycle beginning at 8 AM. Chow pellets and tap water were available *ad libitum* throughout the study. An acclimatization period of 3 days was imposed after delivery by the animal supplier.

The rats were randomly assigned to 1 of 3 groups (12 rats per group): 0.5-mL vehicle (NaCl 0.9%) administered 5 min before ischemia or a single RvD1 dose of 0.1 μg 5 min before ischemia or 5 min after the onset of reperfusion. Vehicle and RvD1 were injected directly into the LV cavity. RvD1 (17(S)-RvD1) was obtained from Cayman Chemical (Ann Arbor, MI, USA) and dissolved in 0.5 mL NaCl 0.9%. Four rats (2 in the vehicle group and 2 in the pre-ischemia group) died during early hours of the reperfusion period and were excluded from the study. All animals were sacrificed 19 days post-MI, after the behavioral tests.

### 3.2. Surgical Procedure

Anesthesia was induced by intraperitoneal ketamine/xylazine injection (60 and 10 mg/kg, respectively). Subsequently, the rats were intubated, and anesthesia was maintained under isoflurane (1%–2%) ventilation. The animals were monitored by electrocardiography throughout the procedure with electrodes placed on their paws. Left thoracotomy at the 5th intercostal space enabled occlusion of the left anterior descending coronary artery with 4-0 silk suture (Syneture; Covidien, Mansfield, MA, USA) and plastic snare, which was confirmed by ST segment alterations and the presence of ventricular sub-epicardial cyanosis. Vehicle or RvD1 was injected into the LV cavity 5 min before ischemia or 5 min after the onset of reperfusion. The suture was removed after 40 min of ischemia, permitting myocardial tissue reperfusion. The thorax was closed with 2-0 silk suture (Syneture; Covidien), and the animals were given a subcutaneous injection of 15,000 IU penicillin G (Duplocillin LA, Intervet Canada Ltd., Whitby, ON, Canada) as well as subcutaneous analgesic injection (2 mg/kg buprenorphine) before being returned to their respective cages.

#### 3.2.1. Behavioral Measures

The tests were selected on the basis of their validity regarding behavioral depression syndrome. All of them were conducted individually, in the morning (between 9 and 11 AM), starting 15 days after surgery. The social interaction test was performed on day 15. The forced swim test was performed on days 16 and 17. All animals were sacrificed on day 19.

#### 3.2.2. Social Interaction Test

Pairs of rats, independently of the assigned experimental group, were placed in a new clean shoebox for 10 min. During this period, 2 observers with no knowledge of the experimental condition monitored 1 animal each; the duration and number of interactions with the other rat were noted. Interactions were scored when one rat smelled, touched the nose or groomed the other rat.

#### 3.2.3. Forced Swim Test

The rats were placed individually in a transparent, 25-cm diameter pool filled with 22–25 °C water to a depth of 30 cm, with no possible escape. One blinded observer timed immobility, swimming and escape trial periods using identical chronometers. The test was conducted for 2 days: day 1 comprised 15 min of habituation, and day 2 entailed the actual 5-min test. On the 2nd day, immobility time was translated into depressive symptoms by comparison to the controls.

#### 3.2.4. Measurement of Infarct Size

On day 19, the rats were restrained in a cone bag and rapidly decapitated. Their hearts were immediately removed and placed in a dish kept on crushed ice. They were removed and washed with saline by retrograde perfusion of the aorta. The left anterior descending coronary artery was occluded at the same site as for MI induction (see above) to map the AR by Evans blue infusion (0.5%). The hearts were frozen (−80 °C for 5 min), sliced into 4 transverse 2-mm sections and placed in 2,3,5-triphenyltetrazolium chloride solution (1%, pH 7.4) at 37 °C for 10 min to better distinguish the area of necrosis (I) from the AR. MI was expressed as percentage of necrosis (I) of the AR ((I/AR) × 100)). In addition, the AR was expressed as percentage of the LV area ((AR/LV) × 100).

### 3.3. Statistical Analysis

The data are reported as means (±SEM). Statistical analyses were performed by SPSS 19 (IBM Corp., Armonk,, NY, USA). Groups were compared by analysis of variance, followed by *post hoc* comparisons (Dunnett test) when significant. If variances were heterogenous, Brown-Forsythe correction was followed by Games-Howell comparisons when applicable. Regression analyses were performed to determine the correlation between MI and immobility time or the socialization time. *p* < 0.05 was considered significant.

## 4. Discussion

The data obtained in this study showed that RvD1, a metabolite of omega-3 PUFA, decreases MI size as well as post-MI depression-like symptoms, assessed in 2 different behavioral tests, whether RvD1 is administered before ischemia or after the onset of reperfusion.

We previously reported that after MI, rats developed depression-like symptoms that can be documented by different behavioral tests [3,4,5,7,8]. These symptoms can be alleviated not only by antidepressants, such as desipramine [21] sertraline or escitalopram [3,4], but also by pentoxifylline [7], a molecule that can reduce the levels of pro-inflammatory cytokines, and by high omega-3 fatty acid diets [6]. Likewise, treatment with a combination of probiotics [5], that shifts the anti- and pro-inflammatory balance towards an anti-inflammatory effect, diminishes depression-like symptoms in the same model. In the present study, we observed that RvD1-treated rats are less immobile in the forced swim test and socialize more than vehicle-treated animals—converging evidence that their depression-like symptoms are reduced.

One important finding of our study is that a single dose of RvD1, administered before or after the onset of reperfusion, is sufficient to attenuate the depression-like symptoms seen 14 days post-MI. This result suggests the possibility that post-MI depression-like symptoms could be reduced if the triggering signal involved in post-MI depression is blocked rapidly. Due to the anti-inflammatory effect of RvD1, we hypothesize that the triggering signal is related to inflammation.

Indeed, inflammation is well-documented after MI, particularly during the first hours of reperfusion [22,23,24,25]. Reperfusion is associated with massive accumulation of neutrophils in ischemic regions [26,27] as well as the release of pro-inflammatory molecules, such as TNF, C5a, a fragment of complement component 5, C-reactive protein, *etc.* [28]. Interestingly, it has been determined that pro-inflammatory molecules, such as IL-1β and TNFα, induce depression-like symptoms, and reduction of their circulating levels could diminish depressive symptoms, suggesting linkage between inflammation and depression [9,10,11]. RvD1 inhibits neutrophil accumulation in ischemic regions [20], indicating an anti-inflammatory effect of this molecule and, due to such an action, we now hypothesize that such properties could explain the antidepressant effects of RvD1 in our model.

We have observed that MI is associated with an increase of apoptotic cells in the limbic system, soon after the onset of reperfusion [10,29]. Apoptosis could be reduced by different interventions, such as antidepressants [3,4] and probiotics [30], and each time that apoptosis is suppressed in our experimental model, depression-like symptoms are diminished, leading to the formulation of our hypothesis that inflammation induces apoptosis in the limbic system and triggers such symptoms. However, more studies are needed to confirm this postulate.

One concern is related to better performance in behavioral tests due to infarct size reduction induced by RvD1. We found a small, but significant correlation between infarct size and immobility time in the forced swim test as well as with socialization time (Figure 4A,B), suggesting that infarct size may explain part of the depressive-like symptoms.

RvD1 reduces infarct size whether it is administered before or after the onset of reperfusion. As we have stated previously, RvD1 possesses cardio-protective properties that could be related to activation of the RISK signaling pathway [20]. Indeed, in a previous study, we observed that Akt activation during the first hours of reperfusion was higher than in vehicle-treated controls [20]. Akt is a major component of a signaling pathway known to be cardio-protective when activated at the onset of reperfusion [31,32]. We and others have previously reported that when the PI3K/Akt pathway is pharmacologically inhibited, cardio-protection offered by CGS21680 or by mechanical post-conditioning is lost [33,34], indicating the central role of Akt in this protective pathway.

We do not know if the mechanism by which RvD1 could activate Akt is direct or indirect, but it could bind 2 G-protein-coupled receptors (ALX and GPR32) [35], and we could speculate that PI3K/Akt activation can occur through them.

## 5. Limitations

One limitation of this study is the absence of sham-operated experimental group. However, we have used sham rats in many previous published studies [3,4,5,7,21,36,37] and results showed that MI was required in order to observe significant effects on behavior.

Another limitation is the route of administration of the treatment: resolvin was administered directly into the left ventricle, which can be considered unusual. This method was selected for three main reasons. First, intraventricular injections avoid metabolization of resolvin before it reaches target regions. Second, less drug is needed and potential side-effects are reduced: in a study aiming to reduce infarct size in a model similar to ours but using peripheral administration of resolvin E1 [38], the amount of drug injected to achieve an infarct size reduction comparable to ours was approximately 300 times higher than the dosage used here. Third, as the heart is already exposed by thoracotomy, injection into the left ventricle is readily accessible compared to the standard IV route, which would have required to canulate a vein and, consequently, possibly affecting the circulatory system integrity. As a matter of fact, angioplasty is increasingly used as a reperfusion procedure [39,40,41,42,43], which permits an easy way to access the left ventricle for injecting drugs via the catheter; this adds ecological validity to our procedure.

## 6. Conclusions

In conclusion, RvD1, an omega-3 PUFA metabolite, given before the ischemic period or after the onset of reperfusion, attenuates post-MI depression-like symptoms and MI size.

## References

[1] Meneses R., Almeida M.C., Abecasis J., Arroja I., Carvalho A., Aleixo A. (2007). Depression in patients with myocardial infarction. Rev. Port. Cardiol..

[2] Frasure-Smith N., Lespérance F., Talajic M. (1993). Depression following myocardial infarction. Impact on 6-month survival. JAMA.

[3] Bah T.M., Benderdour M., Kaloustian S., Karam R., Rousseau G., Godbout R. (2011). Escitalopram reduces circulating pro-inflammatory cytokines and improves depressive behavior without affecting sleep in a rat model of post-cardiac infarct depression. Behav. Brain Res..

[4] Wann B.P., Bah T.M., Kaloustian S., Boucher M., Dufort A.M., le Marec N., Godbout R., Rousseau G. (2009). Behavioural signs of depression and apoptosis in the limbic system following myocardial infarction: Effects of sertraline. J. Psychopharmacol..

[5] Arseneault-Bréard J., Rondeau I., Gilbert K., Girard S.A., Tompkins T.A., Godbout R., Rousseau G. (2012). Combination of *Lactobacillus helveticus* R0052 and *Bifidobacterium longum* R0175 reduces post-myocardial infarction depression symptoms and restores intestinal permeability in a rat model. Br. J. Nutr..

[6] Gilbert K., Arseneault-Breard J., Flores Monaco F., Beaudoin A., Bah T.M., Tompkins T.A., Godbout R., Rousseau G. (2013). Attenuation of post-myocardial infarction depression in rats by n-3 fatty acids or probiotics starting after the onset of reperfusion. Br. J. Nutr..

[7] Bah T.M., Kaloustian S., Rousseau G., Godbout R. (2011). Pretreatment with pentoxifylline has antidepressant-like effects in a rat model of acute myocardial infarction. Behav. Pharmacol..

[8] Rousseau G., Bah T.M., Godbout R., Lakshmanadoss U. (2012). Post-myocardial infarction depression. Novel Strategies in Ischemic Heart Disease.

[9] Francis J., Chu Y., Johnson A.K., Weiss R.M., Felder R.B. (2004). Acute myocardial infarction induces hypothalamic cytokine synthesis. Am. J. Physiol..

[10] Kaloustian S., Bah T.M., Rondeau I., Mathieu S., Lada-Moldovan L., Ryvlin P., Godbout R., Rousseau G. (2009). Tumor necrosis factor-alpha participates in apoptosis in the limbic system after myocardial infarction. Apoptosis.

[11] Van den Biggelaar A.H., Gussekloo J., de Craen A.J., Frolich M., Stek M.L., van der Mast R.C., Westendorp R.G. (2007). Inflammation and interleukin-1 signaling network contribute to depressive symptoms but not cognitive decline in old age. Exp. Gerontol..

[12] Serhan C.N. (2005). Novel eicosanoid and docosanoid mediators: Resolvins, docosatrienes, and neuroprotectins. Curr. Opin. Clin. Nutr. Metab. Care.

[13] Spite M., Serhan C.N. (2010). Novel lipid mediators promote resolution of acute inflammation: Impact of aspirin and statins. Cir. Res..

[14] Serhan C.N., Arita M., Hong S., Gotlinger K. (2004). Resolvins, docosatrienes, and neuroprotectins, novel omega-3-derived mediators, and their endogenous aspirin-triggered epimers. Lipids.

[15] Serhan C.N., Gotlinger K., Hong S., Arita M. (2004). Resolvins, docosatrienes, and neuroprotectins, novel omega-3-derived mediators, and their aspirin-triggered endogenous epimers: An overview of their protective roles in catabasis. Prostaglandins Other Lipid Mediat..

[16] Serhan C.N., Hong S., Gronert K., Colgan S.P., Devchand P.R., Mirick G., Moussignac R.L. (2002). Resolvins: A family of bioactive products of omega-3 fatty acid transformation circuits initiated by aspirin treatment that counter proinflammation signals. J. Exp. Med..

[17] Ohira T., Arita M., Omori K., Recchiuti A., van Dyke T.E., Serhan C.N. (2010). Resolvin e1 receptor activation signals phosphorylation and phagocytosis. J. Biol. Chem..

[18] Lee H.J., Park M.K., Lee E.J., Lee C.H. (2013). Resolvin D1 inhibits TGF-beta1-induced epithelial mesenchymal transition of A549 lung cancer cells via lipoxin A4 receptor/formyl peptide receptor 2 and GPR32. Int. J. Biochem. Cell Biol..

[19] Serhan C.N., Chiang N. (2008). Endogenous pro-resolving and anti-inflammatory lipid mediators: A new pharmacologic genus. Br. J. Pharmacol..

[20] Tran Quang T., Gosselin A.A., Bourque-Riel V., Gilbert K., Charron T., Rousseau G. (2014). Effect of resolvin d1 on experimental myocardial infarction. Exp. Clin. Cardiol..

[21] Wann B.P., Bah T.M., Boucher M., Courtemanche J., le Marec N., Rousseau G., Godbout R. (2007). Vulnerability for apoptosis in the limbic system after myocardial infarction in rats: A possible model for human postinfarct major depression. J. Psychiatry Neurosci..

[22] Simpson P.J., Fantone J.C., Mickelson J.K., Gallagher K.P., Lucchesi B.R. (1988). Identification of a time window for therapy to reduce experimental canine myocardial injury: Suppression of neutrophil activation during 72 h of reperfusion. Circ. Res..

[23] Sharma H.S., Das D.K. (1997). Role of cytokines in myocardial ischemia and reperfusion. Mediat. Inflamm..

[24] Ren G., Dewald O., Frangogiannis N.G. (2003). Inflammatory mechanisms in myocardial infarction. Curr. Drug Targets Inflamm. Allergy.

[25] Nah D.Y., Rhee M.Y. (2009). The inflammatory response and cardiac repair after myocardial infarction. Korean Circ. J..

[26] De Lorgeril M., Rousseau G., Basmadjian A., St-Jean G., Tran D.C., Latour J.G. (1990). Spacial and temporal profiles of neutrophil accumulation in the reperfused ischemic myocardium. Am. J. Cardiovasc. Pathol..

[27] Mullane K.M., Kraemer R., Smith B. (1985). Myeloperoxidase activity as a quantitative assessment of neutrophil infiltration into ischemic myocardium. J. Pharmacol. Methods.

[28] Frangogiannis N.G., Smith C.W., Entman M.L. (2002). The inflammatory response in myocardial infarction. Cardiovasc. Res..

[29] Kaloustian S., Wann B.P., Bah T.M., Girard S.A., Apostolakis A., Ishak S., Mathieu S., Ryvlin P., Godbout R., Rousseau G. (2008). Apoptosis time course in the limbic system after myocardial infarction in the rat. Brain Res..

[30] Girard S.A., Bah T.M., Kaloustian S., Lada-Moldovan L., Rondeau I., Tompkins T.A., Godbout R., Rousseau G. (2009). *Lactobacillus helveticus* and *Bifidobacterium longum* taken in combination reduce the apoptosis propensity in the limbic system after myocardial infarction in a rat model. Br. J. Nutr..

[31] Baxter G.F., Burley D.S. (2008). Reperfusion and calculated risks: Pharmacological postconditioning of human myocardium. Br. J. Pharmacol..

[32] Jonassen A.K., Sack M.N., Mjos O.D., Yellon D.M. (2001). Myocardial protection by insulin at reperfusion requires early administration and is mediated via Akt and p70s6 kinase cell-survival signaling. Circ. Res..

[33] Boucher M., Pesant S., Falcao S., de Montigny C., Schampaert E., Cardinal R., Rousseau G. (2004). Post-ischemic cardioprotection by A2A adenosine receptors: Dependent of phosphatidylinositol 3-kinase pathway. J. Cardiovasc. Pharmacol..

[34] Bopassa J.C., Ferrera R., Gateau-Roesch O., Couture-Lepetit E., Ovize M. (2006). Pi 3-kinase regulates the mitochondrial transition pore in controlled reperfusion and postconditioning. Cardiovasc. Res..

[35] Krishnamoorthy S., Recchiuti A., Chiang N., Yacoubian S., Lee C.H., Yang R., Petasis N.A., Serhan C.N. (2010). Resolvin D1 binds human phagocytes with evidence for proresolving receptors. Proc. Natl. Acad. Sci. USA.

[36] Bah T.M., Wann B.P., Chebli M., le Marec N., Rousseau G., Godbout R. (2007). Insomnia and increased rem sleep pressure in a rat model of post myocardial infarction depression. Can. Sleep Soc..

[37] Bah T.M., Laplante F., Wann B.P., Sullivan R., Rousseau G., Godbout R. (2010). Paradoxical sleep insomnia and decreased cholinergic neurons after myocardial infarction in rats. Sleep.

[38] Keyes K.T., Ye Y., Lin Y., Zhang C., Perez-Polo J.R., Gjorstrup P., Birnbaum Y. (2010). Resolvin E1 protects the rat heart against reperfusion injury. Am. J. Physiol..

[39] Al-Zakwani I., Zubaid M., Al-Riyami A., Alanbaei M., Sulaiman K., Almahmeed W., Al-Motarreb A., Al Suwaidi J. (2012). Primary coronary intervention *versus* thrombolytic therapy in myocardial infarction patients in the middle east. Int. J. Clin. Pharm..

[40] Rymuza H., Kowalik I., Drzewiecki A., Krzyzanowski W., Olszewski M., Dabrowski R., Jedrzejczyk B., Wozniak J., Sosnowski C., Szwed H. (2011). Successful primary coronary angioplasty improves early and long-term outcomes in st segment elevation acute coronary syndromes in patients above 80 years of age. Kardiol. Pol..

[41] Westerhout C.M., Bonnefoy E., Welsh R.C., Steg P.G., Boutitie F., Armstrong P.W. (2011). The influence of time from symptom onset and reperfusion strategy on 1-year survival in st-elevation myocardial infarction: A pooled analysis of an early fibrinolytic strategy *versus* primary percutaneous coronary intervention from captim and west. Am. Heart J..

[42] Gao R.L., Han Y.L., Yang X.C., Mao J.M., Fang W.Y., Wang L., Shen W.F., Li Z.Q., Jia G.L., Lu S.Z. (2010). Thorombolytic therapy with rescue percutaneous coronary intervention *versus* primary percutaneous coronary intervention in patients with acute myocardial infarction: A multicenter randomized clinical trial. Chin. Med. J..

[43] Borgia F., Goodman S.G., Halvorsen S., Cantor W.J., Piscione F., le May M.R., Fernandez-Aviles F., Sanchez P.L., Dimopoulos K., Scheller B. (2010). Early routine percutaneous coronary intervention after fibrinolysis *vs.* Standard therapy in ST-segment elevation myocardial infarction: A meta-analysis. Eur. Heart J..

